# Human papillomavirus genotype distribution and cervical squamous intraepithelial lesions among high-risk women with and without HIV-1 infection in Burkina Faso

**DOI:** 10.1038/sj.bjc.6603252

**Published:** 2006-07-11

**Authors:** M-N Didelot-Rousseau, N Nagot, V Costes-Martineau, X Vallès, A Ouedraogo, I Konate, H A Weiss, P Van de Perre, P Mayaud, M Segondy

**Affiliations:** 1 Department of Virology, Montpellier University Hospital, 34295 Montpellier, France; 2 Laboratory of Virology, UMR145 (University of Montpellier and Institut de Recherche pour le Développement), Saint-Eloi Hospital, 80 Augustin Fliche Ave., 34295 Montpellier Cedex 5, France; 3 Centre Muraz, BP153, Bobo Dioulasso, Burkina Faso; 4 London School of Hygiene and Tropical Medicine, Keppel Street, London WC 1E 7HT, UK; 5 Department of Pathology, Montpellier University Hospital, 34295 Montpellier, France

**Keywords:** HPV, genotypes, SIL, HIV-1, Burkina Faso, Africa

## Abstract

Human papillomavirus (HPV) infection and cervical squamous intraepithelial lesions (SILs) were studied in 379 high-risk women. Human papillomavirus DNA was detected in 238 of 360 (66.1%) of the beta-globin-positive cervical samples, and 467 HPV isolates belonging to 35 types were identified. Multiple (2–7 types) HPV infections were observed in 52.9% of HPV-infected women. The most prevalent HPV types were HPV-52 (14.7%), HPV-35 (9.4%), HPV-58 (9.4%), HPV-51 (8.6%), HPV-16 (7.8%), HPV-31 (7.5%), HPV-53 (6.7%), and HPV-18 (6.4%). Human immunodeficiency virus type 1 (HIV-1) seroprevalence was 36.0%. Human papillomavirus prevalence was significantly higher in HIV-1-infected women (87 *vs* 54%, prevalence ratio (PR)=1.61, 95% confidence interval (CI): 1.4–1.8). High-risk HPV types (71 *vs* 40%, PR=1.79, 95% CI: 1.5–2.2), in particular HPV-16+18 (22 *vs* 9%, PR=2.35, 95% CI: 1.4–4.0), and multiple HPV infections (56 *vs* 23%, PR=2.45, 95% CI: 1.8–3.3) were more prevalent in HIV-1-infected women. High-grade SIL (HSIL) was identified in 3.8% of the women. Human immunodeficiency virus type 1 infection was strongly associated with presence of HSIL (adjusted odds ratio=17.0; 95% CI 2.2–134.1, *P*=0.007) after controlling for high-risk HPV infection and other risk factors for HSIL. Nine of 14 (63%) HSIL cases were associated with HPV-16 or HPV-18 infection, and might have been prevented by an effective HPV-16/18 vaccine.

Cervical cancer is the most frequent cancer of women in developing countries, particularly in sub-Saharan Africa (Parkin *et al*, 1999), and it is now well established that genital infection with certain types of human papillomavirus (HPV) causes virtually all cases of cervical intraepithelial neoplasia (CIN) and invasive cervical cancer (ICC) (Walboomers *et al*, 1999). The HPV types that infect the genital tract have been subdivided into low-risk (LR) types, which are principally found in nonmalignant lesions such as genital warts, and high-risk (HR) types, which are associated with the development of CIN and ICC. Human papillomavirus types 6, 11, 40, 42, 43, 44, 54, 61, 70, 72, 81, and 89 are classified as LR types, whereas types 16, 18, 31, 33, 35, 39, 45, 51, 52, 56, 58, 59, 68, 73, and 82 are classified as HR types. In addition, HPV types 26, 53, and 66 are considered as probably carcinogenic (Muñoz *et al*, 2003).

Human papillomavirus prophylactic vaccines are now being developed and promising results have been obtained with recombinant L1 capsid protein virus-like particles (VLPs). However, the current HPV vaccines target the most prevalent high-risk HPV (HR-HPV) types worldwide, namely HPV-16 and HPV-18 (Koutsky *et al*, 2002; Ault *et al*, 2004; Brown *et al*, 2004; Harper *et al*, 2004). It has been shown that crossneutralisation induced by L1 VLPs represents less than 1% of the neutralising activity induced by the dominant conformational epitope (Combita *et al*, 2002), indicating that current HPV vaccines would be able to confer only type-specific immunity. Therefore, effectiveness of these vaccines on the prevention of cancer may be lower in populations highly affected by HR-HPV types other than HPV-16 and HPV-18. Thus, it is important to document the distribution of HPV genotypes in HPV-infected women and in women with cervical neoplasia in African countries in order to assess the potential effectiveness of a bivalent HPV-16/18 vaccine.

There have been few detailed studies of HPV genotypes and their association with intraepithelial lesions or ICC in sub-Saharan Africa. Available data suggest, however, a higher prevalence and wider spectrum of oncogenic HPV types compared to studies conducted elsewhere (Castellsague *et al*, 2001; De Vuyst *et al*, 2003; Mayaud *et al*, 2003; Clifford *et al*, 2005; Wall *et al*, 2005). Furthermore, the high background prevalence of human immunodeficiency virus type 1 (HIV-1) observed in many African settings adds complexity to our understanding of the epidemiology of HPV and cervical neoplasia. Studies conducted in industrialised countries have shown that HIV-1 alters the natural history of HPV infection by upregulating HPV persistence and recurrences, thereby facilitating progression to high-grade lesions and cancer (Sun *et al*, 1997; Ahdieh *et al*, 2001; Strickler *et al*, 2005). Consequently, cervical cancer has been included in the list of AIDS-defining opportunistic conditions by the Centres for Disesase Control and Prevention (Centers for Disease Control and Prevention, 1993). However, the relationships between HIV-1, HPV and squamous intraepithelial lesions (SIL) in sub-Saharan Africa are unclear. Some studies have found no association between HIV-1 and HPV (Mayaud *et al*, 2001), others found an association between HIV-1 and HPV, but no association between HIV-1 and SIL (De Vuyst *et al*, 2003), while others found a strong association between HIV-1, HR-HPV, high-grade SIL (HSIL), and cervical cancer (La Ruche *et al*, 1998; Chirenje *et al*, 2002; Hawes *et al*, 2003). Surprisingly, the relentless progression of the AIDS epidemic on the African continent has not been paralleled by increases in cervical cancer rates, in contrast with rates of other AIDS-linked cancers, such as Kaposi's sarcoma or non-Hodgkin's lymphomas (Parkin *et al*, 1999). This may be due, in part, to a much-reduced life expectancy among HIV-1-infected women in Africa contrasting with a long duration for ICC development. Further understanding of this relationship is essential in order to assess the possible response to a newly introduced HPV-16/18 vaccine in such populations.

In Burkina Faso, a country in West Africa with an estimated HIV-1 prevalence among the general adult population of 4.2% (http://www.unaids.org), virtually nothing is known of the epidemiology of HPV and HPV-related malignancies, and the impact of HIV on these conditions. The aim of the present study was to determine the prevalence of HPV infection, the distribution of HPV genotypes, the frequency of SIL, and their relationships with HIV-1, in a group of high-risk women in Bobo Dioulasso, the second largest city of Burkina Faso.

## MATERIALS AND METHODS

### Study population and sample collection

We performed a cross-sectional survey nested within the Yerelon cohort study, a research programme that aims to design, implement, and evaluate interventions reducing the risk of HIV-1 and other sexually transmitted infections (STI) among female sex workers (FSWs). The cohort inclusion process has been described elsewhere (Nagot *et al*, 2002). In brief, FSWs were informally approached at their place of work (streets, bars, or home) through an existing network of trained peer educators, and women were referred to the Centre Municipal d'Hygiene, a public health facility in Bobo Dioulasso. After extensive information and a reflection period of at least 2 weeks, women consenting to participate were invited to answer a standardised questionnaire eliciting information on sociodemographic factors, sexual behaviour, past and current sexual health. Women were examined with a speculum during which genital samples were collected for the diagnosis of vaginal and cervical infections. Blood samples were drawn for HIV, syphilis and Herpes simplex virus type 2 (HSV-2) serologies. Participants were followed up every 4 months with serological re-testing as appropriate.

Between December 2003 and March 2004, consecutive women taking part in the cohort study were requested permission for an additional cervical swab for liquid-based cytology and HPV-DNA detection using a Cervex brush (Cytyc, Montrouge, France). The brush was stirred into a vial of PreservCyt transport solution (Cytyc) and kept at room temperature until shipment to the Pathology Laboratory at the University Hospital, Montpellier, France. The Yerelon Cohort Research Programme has been approved by the Ethical Committee of the Centre Muraz, Bobo Dioulasso, the National Ethical Committee of the Ministry of Health, Burkina Faso, and the Ethics Committee of the London School of Hygiene and Tropical Medicine.

### Cervical cytology

Thin-layer cytological smears were prepared from the Cytic cell suspension vial by controlled membrane transfer technology using the ThinPrep 2000 processor (Cytyc). After Papanicolaou (Pap) staining, slides were independently examined by two senior pathologists who were blinded to all other study results. The 2001 Bethesda classification (Smith, 2002) was used for slide interpretation.

### HPV detection and typing

DNA was extracted from the residual cell suspension as described previously (Gravitt *et al*, 2000). Samples were tested for the presence of amplifiable DNA and absence of PCR inhibitors by performing beta-globin PCR (Saiki *et al*, 1988). Two single-step PCRs using consensus primer pairs were performed on the beta-globin-positive samples using first the primer pair MY09/MY11, which allows amplification of a 450 bp fragment in the HPV L1 gene (Manos *et al*, 1989); negative samples were tested using the primer pair GP5+/GP6+, which allows amplification of a 140 bp fragment in L1 (de Roda Husman *et al*, 1995) and the samples found negative by both MY09/MY11- and GP5+/GP6+-mediated single-step PCRs were tested by nested PCR using the primers MY09/MY11 in the first round and the primers GP5+/GP6+ in the second round. The MY09 primer mixture contained the HPV-51-specific HMB01 oligonucleotide as described previously (Gravitt *et al*, 2000). The amplification mixtures contained 50 pmol of each primer in the presence of 1 × PCR buffer, 6 mM (MY09/MY11 primers), or 4 mM (GP5+/GP6+ primers) MgCl_2_, 200 *μ*mol (each) dNTPs, 1.5 U of AmpliTaq gold DNA polymerase (Applied Biosystems, Courtaboeuf, France), and 5 *μ*l of DNA extract in a volume of 50 *μ*l. The PCR conditions were 95°C for 9 min, followed by 40 cycles of 95°C for 1 min, 55°C (MY09/MY11 primers) or 38°C (GP5+/GP6+ primers) for 1 min and 72°C for 1 min, and a final extension at 72°C for 5 min. For the nested PCR, 2 *μ*l of the first-round product were used in the second-round PCR.

HPV typing of the HPV DNA-positive samples was performed using the INNO-LiPA HPV Genotyping v2 test (Innogenetics, Ghent, Belgium). This test is based on the amplification of a part of the L1 region of the HPV genome using the broad-spectrum SPF_10_-biotinylated primers. Biotinylated amplicons are subsequently hybridised with HPV type-specific oligonucleotide probes, which are immobilised as parallel lines on membrane strips. After hybridisation and stringent washing, streptavidin-conjugated alkaline phosphatase is added and bound to any biotinylated hybrid formed. Incubation with BCIP/NBT chromogen yields a purple precipitate and the results can be visually interpreted (Kleter *et al*, 1999). This test allows detection of 24 HPV types, namely HR-HPV types 16, 18, 31, 33, 35, 39, 45, 51, 52, 56, 58, 59, and 68, LR-HPV types 6, 11, 40, 42, 43, 44, 54, 70, and 74, and the probably oncogenic HPV types 53 and 66.

In addition, HPV typing was performed by sequencing and phylogenetic analyse on HPV DNA-positive samples that could not be typed using the INNO-LiPA Genotyping v2 test. The PCR products obtained using the GP5+/GP6+ primers were directly sequenced with the BigDye Terminator cycle sequencing kit v3.1 (Applied Biosystems) on the ABI PRISM 3100 genetic analyser (Applied Biosystems); GP5+ and GP6+ primers served as forward and reverse primers, respectively. The nucleotide sequences were aligned and compared with those of known HPV types available through the GenBank database (http://www.ncbi.nlm.nih.gov/) by use of the CLUSTAL W multiple sequence alignment program. Phylogenetic trees were constructed by the neighbour-joining method and reliability of the branching orders was assessed by the bootstrap approach with CLUSTAL W. A nucleotide sequence was assigned to a HPV type if it displayed greater than 90% homology with this type (de Villiers *et al*, 2004).

### Other reproductive tract infection (RTI) or STI

Vaginal smears were examined microscopically for the presence of *Trichomonas vaginalis*, *Candida* spp, and bacterial vaginosis (using the Nugent's score on a Gram-stained smear; Nugent *et al*, 1991). One cervical swab was cultured onto modified Thayer–Martin media plates using standard procedures to detect the presence of *Neisseria gonorrhoeae*. Molecular diagnosis of *Chlamydia trachomatis* was not available at the time of the survey, but this infection had been shown to be rare by PCR (<2%) in this population (Nagot *et al*, 2004). Venous blood samples were tested for: HIV-1 and -2 serologies, using a validated national strategy based on two independent ELISA tests (Meda *et al*, 1999); syphilis serology, by rapid plasma reagin test (RPR, Human GmbH, Wiesbaden, Germany) with reactive sera confirmed by *Treponema pallidum* haemagglutination assay (TPHA, Newmarket Laboratories Ltd, Kentford, UK); and HSV-2 serology, using a specific gG2-ELISA test (Kalon HSV-2, Kalon Biologicals, Aldershot, UK) proved to have a good sensitivity and specificity on African sera (Van Dyck *et al*, 2004). CD4 lymphocyte counts were measured from blood samples using a standard flow cytometric method.

### Data analysis

Data were analysed using STATA 8.1 (StataCorp, College Station, TX, USA). Associations between HIV and HPV were assessed with prevalence ratios (PR) and their 95% confidence intervals (95% CI). Associations between HSIL and exposures were assessed with odds ratios (OR) and 95% CI, as HSIL was a rare event, and statistically tested by Fisher's exact test and *χ*^2^ for trend where appropriate. Adjusted ORs (AOR) and corresponding 95% CI were calculated by multiple logistic regression for factors significantly associated with HSIL in univariate analysis (i.e., with *P*-values <0.1, or crude OR ≥2 or <0.5), or for known risk factors for HSIL reported in the literature (e.g., young age at first sex, smoking, hormonal contraception, presence of other STIs, or low CD4 cell count). Associations of prevalence of HPV types with cervical lesions categories were tested by *χ*^2^ for trend.

## RESULTS

### Study population characteristics, HIV-1, and RTI/STI prevalence

A total of 379 women were seen during the study period and all agreed to participate. Their median age was 28.0 years (range 16–54 years) and the mean age at first sexual intercourse was 16.7 years. Participants had been involved in commercial sex for a median of 3.2 years, with a median number of 3.3 weekly sexual partners. Condoms were reportedly used in all sexual encounters with first-time clients by 77.1% of women, but were seldom used with their regular partner. Only 12% of participants used hormonal contraception and 7% were current smokers. Prevalence of bacterial vaginosis, *C. albicans*, and *T. vaginalis* were 42.4, 6.8, and 9.6% respectively, and no diagnosis of *N. gonorrhoeae* was made. Only two cases of serological syphilis (RPR- and TPHA-positive sera) were detected. Human immunodeficiency virus type 1 and HSV-2 seroprevalence were 36.6 and 69.8%, respectively.

### HPV detection and typing

**Figure 1:**
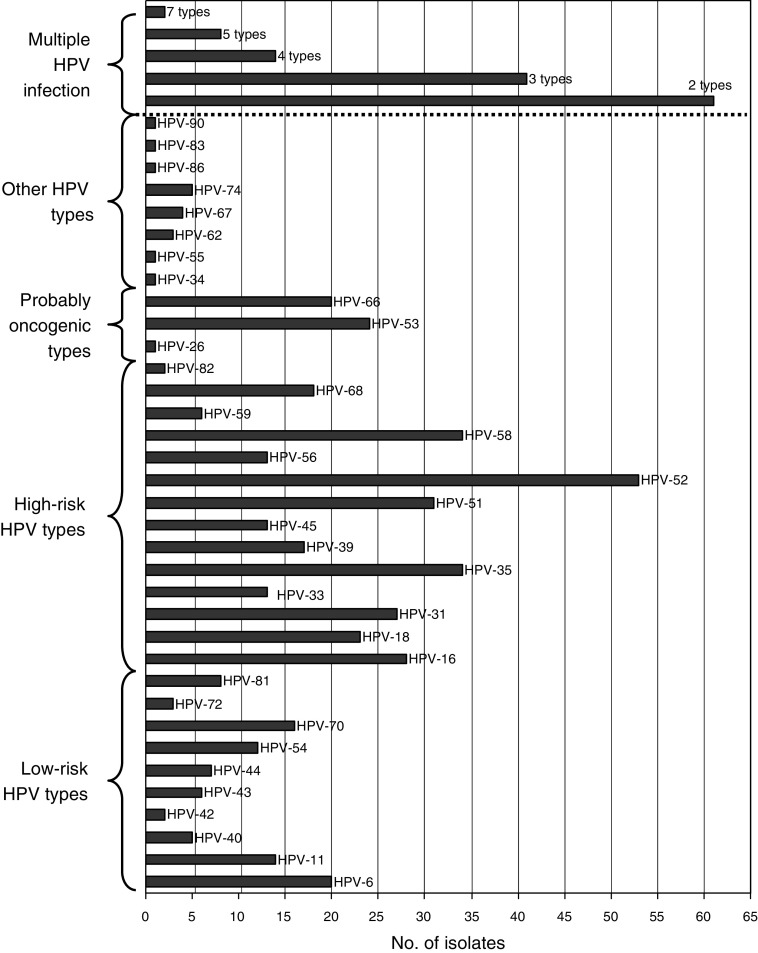
Distribution of HPV types identified among 349 high-risk women in Bobo-Dioulasso, Burkina Faso.

Cervical samples were obtained from all 379 women, and 360 (95.0%) were positive for beta-globin DNA. The prevalence of cervical HPV was 66.1% (238 of 360): 114 samples were positive with the MY09/MY11 primer pair, 50 were positive with the GP5+/GP6+ primer pair, and 74 were positive by nested PCR. Human papillomavirus types could be identified by LiPA in 211 samples and by sequencing in the remaining 27 samples. A total of 467 HPV infections belonging to 35 types were identified (Figure 1). The most prevalent HPV types were HPV-52 (14.7%), HPV-35 (9.4%), HPV-58 (9.4%), HPV-51 (8.6%), HPV-16 (7.8%), HPV-31 (7.5%), HPV-53 (6.7%), and HPV-18 (6.4%). Multiple HPV infections (2–7 types) were identified in 126 of 360 (35.0%) women and accounted for 52.9% (126 of 238) of HPV-infected women.

### Cervical cytology

Interpretable cervical smears were obtained from 366 (97%) women. Squamous intraepithelial lesion were detected in 88 (24.0%) women, with 74 (20.2%) being classified as LSIL and 14 (3.8%) as HSIL. Thirteen (3.5%) samples were classified as atypical squamous cells of undetermined significance (ASCUS).

### Relationships between HIV-1, HPV infection, and cervical SIL

A total of 350 women had a complete set of data available, including an interpretable Pap smear, a positive beta-globin DNA PCR and known behavioural and demographic characteristics. Among these women, 126 (36.0%) were infected with HIV-1 and 30 (23.8%) of them had a CD4 cell count <200 cells *μ*l^−1^. One woman, infected with HIV-2 alone (and infected with HPV-70) was excluded from further analyses. Subsequent analyses are based on the 349 remaining women.

**Figure 2:**
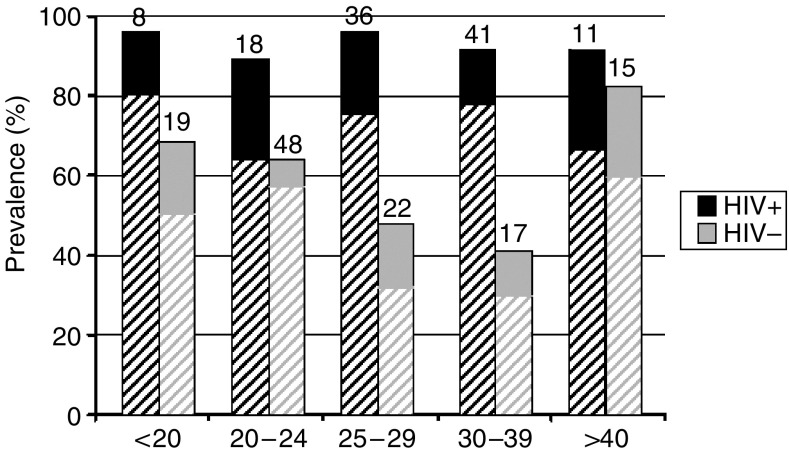
Distribution of HPV infection according to age and HIV-1 status among 349 women in Bobo Dioulasso, Burkina Faso. Numbers of women in groups are indicated at the top of bars. The hatched part of the bar represents the proportion of women infected with at least one high-risk HPV type, whereas the unicoloured part represents the proportion of women infected with low-risk HPV types only.

As shown in Table 1, the prevalence of HPV infection was significantly higher in HIV-1-infected women, mainly owing to a higher prevalence of HR-HPV, including HPV types 16 and 18. The prevalence of multiple HPV infection was also increased in HIV-1-infected women. Age-related HPV prevalence according to HIV-1 serostatus is shown in Figure 2. Among HIV-1-seronegative women, HR-HPV infection varied significantly, being more frequent in the younger and older age groups (*P*=0.03). In contrast, there was no statistically significant association of HPV with age among HIV-1-seropositive women (*P*=0.9).

Despite the participants' young age, a high prevalence (24.9%) of cervical cytological abnormalities was observed (Table 2). As expected, SIL was more prevalent among the HPV-positive women (*P*<0.001), and among women with multiple HPV infections compared to those infected with a single HPV type (54 of 123 (43.9%) *vs* 24 of 109 (22.0%), *P*<0.001). Among HPV-infected women, prevalence of SIL was significantly higher among those co-infected with HIV-1 (*P*<0.001). HSIL was observed exclusively among HPV-infected women, and all but one HSIL cases were observed among HIV-1-infected women (*P*<0.001) (Table 2). Only HIV-1 infection and HR-HPV types were statistically significantly associated with HSIL in univariate analysis (Table 3), while low CD4 cell count (<200 cells *μ*l^−1^) had an association of borderline significance. None of the classical cofactors of HSIL (e.g., age at fist sex, smoking, presence of other STIs) reported in the literature were associated with HSIL in this study, in part because of the small number of HSIL cases and/or the low prevalence of some of these variables. In multivariate analysis, only HIV-1 infection remained strongly associated with the presence of HSIL (AOR=17.0, 95% CI: 2.2–134.1, *P*=0.007), while there was suggestion for a nonstatistically significant association between HR-HPV and HSIL (AOR=7.0, 95% CI 0.9–55.6, *P*=0.07).

To explore the potential impact of a bivalent HPV-16/18 vaccine, we have examined the prevalence of HPV-16- and HPV-18-related infections according to HIV-1 serostatus and cervical lesions categories (Table 4). Human papillomavirus-16 was the most prevalent (42.9%) genotype among the 14 women with HSIL, while HPV types 16 or 18 were found in nine (64.3%) of these women. All but one woman with HSIL – who was infected with HPV types 54 and 66 – were infected with HPV types belonging to HPV-16- or HPV-18-related phylogenetic groups.

## DISCUSSION

This is the first report of an epidemiological study of cervical HPV and SIL in Burkina-Faso. We did not conduct a general population-based study but decided to target a population of highly sexually exposed women. Such women are likely to concentrate the majority of HPV types circulating in the general population of the region, thereby providing some of the information required to assess the usefulness of an HPV vaccine. Moreover, our choice of study population allowed, with an economical sample size, exploration of the relationships between HPV subtypes, presence of SIL, and the role played by HIV in promoting persistence of HPV and progression to HSIL. This is critical information since, ultimately, an HPV vaccine's effectiveness will depend upon the proportion of HSIL and ICC cases attributable to the subtypes included in the vaccine, and possibly the background HIV prevalence in the population, which might act as a catalyst in the upregulation of certain HPV types. Moreover, in some sub-Saharan settings, it is conceivable that HSIL and ICC may disproportionately be found among young HIV-positive women. Thus, although the epidemiological picture found in our study population might not be indicative of infection levels in the general population of Burkina Faso, the diversity of HPV types found and their association with cervical lesions would constitute valid and important data to inform HPV vaccine strategies in the country. Finally, our study represents one of the most detailed and exhaustive molecular studies of HPV in sub-Saharan Africa, which is an important prerequisite in describing HPV epidemiology. Indeed, we detected HPV DNA in cervical samples using the two most commonly used consensus PCR primer sets for genital HPV, namely MY09/MY11 and GP5+/GP6+ (Qu *et al*, 1997), and samples found negative by both PCR reactions were tested by nested PCR. Positive HPV samples were typed by LiPA and by sequencing when LiPA was inconclusive, leaving no sample unidentified.

While the epidemiology of genital HPV types has been relatively better studied in Southern and Eastern African countries such as Kenya (Temmerman *et al*, 1999; De Vuyst *et al*, 2003), Malawi (Miotti *et al*, 1996), Mozambique (Castellsague *et al*, 2001), Tanzania (Mayaud *et al*, 2003), Uganda (Serwadda *et al*, 1999), and Zimbabwe (Gravitt *et al*, 2002; Baay *et al*, 2004), data from Western Africa have been scarce. The prevalence of HPV infection and associated cervical lesions have been studied in Ivory Coast (La Ruche *et al*, 1998) and Mali (Bayo *et al*, 2002), but detailed HPV types were only reported from Senegal (Xi *et al*, 2003), Nigeria (Thomas *et al*, 2004), and The Gambia (Wall *et al*, 2005). Prevalence rates of HPV infection ranging from 13 to 40% have been reported from low-risk or general populations of sub-Saharan African countries (Miotti *et al*, 1996; Temmerman *et al*, 1999; Castellsague *et al*, 2001; De Vuyst *et al*, 2003; Mayaud *et al*, 2003; Xi *et al*, 2003; Baay *et al*, 2004; Thomas *et al*, 2004; Wall *et al*, 2005). The 66% prevalence found in the present study, which is still much higher than the 40% prevalence found in similar high-risk populations in Africa (Kreiss *et al*, 1992; Langley *et al*, 1996; Piper *et al*, 1999), might reflect the high sexual exposure of our study population and, as mentioned earlier, may be attributed in part to the exhaustive nature of our HPV detection strategy. Indeed, the use of a primer pair alone to detect HPV by PCR may be responsible for the underestimation of infections with certain HPV types. For example, HPV types 26, 35, 42, 45, 52, 54, 55, 59, 66, 68, 73, and 83 may be missed by using only the MY09/MY11 primer pair (Qu *et al*, 1997; Gravitt *et al*, 2000). Human papillomavirus-52, HPV-35, and HPV-58 were the most prevalent types in the present study. A high prevalence of these HPV types has been also observed in other African studies (Gravitt *et al*, 2000; Castellsague *et al*, 2001; De Vuyst *et al*, 2003; Mayaud *et al*, 2003; Xi *et al*, 2003; Baay *et al*, 2004; Thomas *et al*, 2004; Wall *et al*, 2005).

The higher HR-HPV prevalence observed in the younger (<25 years) and older (>40 years) HIV-1-seronegative women compared to the middle age groups is consistent with high rates of HPV acquisition following entry into sexually active life, and high rates of recurrence or persistence associated with older ages, as described elsewhere (Castle *et al*, 2005). Although HR-HPV infection rates were much higher, there was no statistically significant association with age among the HIV-1-positive women, possibly reflecting the process of upregulation and persistence of HPV elicited by HIV-1, as suggested by others (Palefsky *et al*, 1999; Ahdieh *et al*, 2001; Moscicki *et al*, 2004b), independent of age.

A high prevalence of HIV-1 infection was observed in our study population, and HIV-1 infection was associated with an increased rate of HPV infection, mainly restricted to HR-HPV types. This finding may result from the high level of sexual exposure to both viruses as well as, as mentioned above, from persistence or recurrence of oncogenic HPV following HIV-1 induced immunosuppression. We also found that HIV-1 infection was independently associated with an increase in SIL among HPV-positive women and that HIV-1 represented an important risk factor for the presence of HSIL. These findings are in agreement with other African series (La Ruche *et al*, 1998; Chirenje *et al*, 2002; Hawes *et al*, 2003) and with many studies in industrialised countries (Sun *et al*, 1997; Ahdieh *et al*, 2001; Moscicki *et al*, 2004b; Strickler *et al*, 2005).

More than half of the HPV-infected FSWs presented multiple HPV infection, which was associated with higher HIV-1 and SIL prevalence. Previous studies have reported a high prevalence of multiple HPV infections among HIV-infected women (Palefsky *et al*, 1999; Gravitt *et al*, 2002) which is associated with an increased risk of intraepithelial neoplasia and of cancer (Moscicki *et al*, 2004a; Herrero *et al*, 2005).

As was found in other African studies (Ter Meulen *et al*, 1992; Chabaud *et al*, 1996; La Ruche *et al*, 1998; Bayo *et al*, 2002; Kay *et al*, 2003), HPV-16 and -18 were less common than other HR-HPV types overall, but they were involved in 64% of HSIL lesions, while 93% of women with HSIL had viruses related to the HPV-16 or HPV-18 phylogenetic groups. Therefore, administration of a bivalent HPV-16/18 vaccine in this population before they get exposed might have the potential to prevent the majority of high-grade lesions in this population, but this would need to be determined in prospective studies. It is not known, however, whether successful control of HPV-16 or -18 might not lead to replacement by other oncogenic HPV types, which are highly prevalent in our population as in many parts of sub-Saharan Africa (Muñoz *et al*, 2004).

In conclusion, our study confirms the high diversity of HPV genotypes, the burden of HPV-related cervical lesions, the role of HIV-1 as a cofactor of HPV persistent infection, multiple-type infection, and SIL in highly sexually exposed young African women. The potentially preventable nature of prevalent HPV types constitutes a compelling argument to propose HPV-16/18 vaccination to such highly vulnerable populations. However, the safety, immunogenicity and impact of these vaccines in populations with high background HIV-1 prevalence, remain to be determined.

## Figures and Tables

**Table 1:** Infection with high-risk or low-risk HPV types according to HIV-1 status, among 349 high-risk women in Bobo Dioulasso, Burkina Faso

	**HIV**-**1 status**		
	**Positive (*n*=126)**	**Negative (*n*=223)**	
**HPV types**	**No. (%)**	**No. (%)**	**Prevalence ratio (95% CI)**
HPV-positive	110 (87)	121 (54)	1.61 (1.4–1.8)
At least one high-risk HPV type	89 (71)	89 (40)	1.79 (1.5–2.2)
Infection with HPV-16 and/or -18	28 (22)	21 (9)	2.35 (1.4–4.0)
Low-risk HPV type only	21 (17)	32 (14)	1.12 (0.8–1.6)
Multiple HPV infections	71 (56)	51 (23)	2.45 (1.8–3.3)

CI=confidence interval; HIV=human immunodeficiency virus; HPV=human papillomavirus.

**Table 2:** Prevalence of SILs according to HPV and HIV-1 status among 349 high-risk women in Bobo Dioulasso, Burkina Faso

	**No. (%) of cervical lesions^a^**		
**HPV and HIV**-**1 status**	**LSIL**	**HSIL**	**All SIL**
HPV− HIV-1− (*n*=102)	7 (6.9)	0 (0.0)	7 (6.9)
HPV− HIV-1+ (*n*=16)	2 (12.5)	0 (0.0)	2 (12.5)
HPV+ HIV-1− (*n*=121)	18 (14.9)	1 (0.8)	19 (15.7)
HPV+ HIV-1+ (*n*=110)	46 (41.8)	13 (11.8)	59 (53.6)
All (*N*=349)	73 (20.9)	14 (4.0)	87 (24.9)

HIV=human immunodeficiency virus; HPV=human papillomavirus; HSIL=high-grade SIL; LSIL=low-grade SIL; SIL=squamous intraepithelial lesions.

a Atypical squamous cells of undetermined significance (ASCUS) are not included.

**Table 3:** Univariate analysis: associations between HSIL and sociodemographic, behavioural, and biological factors among 349 high-risk women in Bobo Dioulasso, Burkina Faso

**Characteristics^a^**	**HSIL/total (%) (14/349)**	**Crude OR (95% CI)**	***P*-value for Fisher's exact test or for test for trend^b^**
*Sociodemographic factors*			
Age (years)	(*n*=349)		
15–24	0/144 (0)	NC	0.001
25–34	9/137 (7)	1	0.09
35–39	4/34 (11)	1.67 (0.5–5.8)	0.05
>40	1/29 (3)	0.49 (0.06–4.1)	1
Parity	(*n*=300)		
Nulliparous	1/52 (2)	1	
Parity 1–3	7/177 (4)	0.48 (0.05–4.0)	
Parity 4+	5/66 (7)	0.26 (0.03–2.3)	0.4^*^
			
*Behavioural factors*			
Age at first sex (years)	(*n*=323)		
>18	2/13 (15)	1	
15–18	9/207 (4)	0.25 (0.05–1.3)	
⩽15	2/103 (2)	0.11 (0.01–0.9)	0.06^*^
No. years in sex work	(*n*=311)		
<2	3/159 (2)	1	
≥2	9/152 (6)	3.27 (0.9–12.4)	0.08
Smoking	(*n*=348)		
No	13/322 (4)	1	
Yes	1/26 (4)	0.95 (0.1–7.6)	1
			
*Biological factors*			
Hormonal contraception	(*n*=258)		
No	8/211 (4)	1	
Yes	3/47 (6)	1.73 (0.4–6.8)	0.4
HR-HPV types	(*n*=349)		
Not present	1/171 (0.6)	1	
Present	13/178 (7.3)	13.4 (1.7–107.1)	0.002
*T. vaginalis*	(*n*=299)		
Not present	10/272 (4)	1	
Present	0/29 (0)	NC	0.6
HSV-2 serology	(*n*=347)		
Negative	3/106 (3)	1	
Positive	11/241 (4)	1.4 (0.4–6.0)	0.6
HIV-1 serology	(*n*=349)		
Negative	1/223 (0.4)	1	
Positive	13/126 (10)	25.5 (3.1–211.1)	<0.001
CD4 count (in HIV+)	(*n*=113)		
≥200 cells *μ*l^−1^	5/86 (6)	1	
<200 cells *μ*l^−1^	5/27(19)	3.68 (0.9–14.3)	0.06

CI=confidence interval; HIV=human immunodeficiency virus; HPV=human papillomavirus; HR-HPV=high-risk HPV genotypes; HSIL=high squamous intraepithelial lesions; NC=odds ratio (OR) not calculated as cell value=0.

a Different denominators for some characteristics, depending on questionnaire structure, and some missing data.

b Test for trend.

**Table 4:** Infection with HPV-16 and -18 related types according to HIV-1 status and Pap smear result among 349 women in Bobo Dioulasso, Burkina Faso

	**HIV-1 status**									
	**Positive (*n*=126)**					**Negative (*n*=223)**				
	**No. (%) with cytological result**					**No. (%) with cytological result**				
**HPV status**	**HSIL (*n*=13)**	**LSIL (*n*=48)**	**ASCUS (*n*=2)**	**Normal (*n*=63)**	***P*-value^a^**	**HSIL (*n*=1)**	**LSIL (*n*=25)**	**ASCUS (*n*=10)**	**Normal (*n*=187)**	***P*-value^a^**
HPV-16 only	5 (38)	6 (13)	1 (50)	1 (2)	<0.001	1 (100)	2 (8)	0 (0)	11 (6)	0.001
HPV-18 only	3 (23)	7 (15)	0 (0)	6 (10)	0.5	0 (0)	1 (4)	1 (10)	5 (3)	0.6
HPV-16 or -18	8 (62)	12 (25)	1 (50)	7 (11)	0.001	1 (100)	3 (12)	1 (10)	17 (9)	0.03
HPV-16 group^b^	10 (77)	33 (69)	2 (100)	25 (40)	0.003	1 (100)	12 (48)	5 (50)	47 (25)	0.02
HPV-18 group^c^	5 (38)	17 (35)	1 (50)	19 (30)	0.8	0 (0)	5 (20)	3 (30)	23 (12)	0.3
HPV-16 group or HPV-18 group	12 (92)	39 (81)	2 (100)	33 (52)	0.001	1 (100)	15 (60)	6 (60)	62 (33)	0.01

ASCUS=atypical squamous cells of undetermined significance; HIV=human immunodeficiency virus; HPV=human papillomavirus; HR=high-risk types; HSIL=high-grade squamous intraepithelial lesions; LR=low-risk types; LSIL=low-grade squamous intraepithelial lesions.

a *χ*^2^ for trend over cervical abnormalities categories.

b HPV-16 phylogenetic group includes the following HPV sub-types: 16, 31, 33, 35, 52, 58, and 67.

c HPV-18 phylogenetic group includes the following HPV sub-types: 18, 39, 45, 59, 68, and 70.

## Appendix A

The Yerelon Study Group is composed of the following persons: Eloi Bahembera, Minata Coulibaly, Marie-Christine Defer, Ramata Diallo, Didier Djagbare, Djeneba Drabo, Issouf Konate, Florent Ky-Dama, Gilles M'Boutiki, Nicolas Meda, Ines Millogo, Nicolas Nagot, Abdoulaye Ouedraogo, François Rouet, Anselme Sanon, Haoua Sawadogo, Roselyne Vallo, and Laurence Vergne (Centre Muraz, Bobo Dioulasso, Burkina Faso); Jean-Baptiste Andonaba and Adrien Sawadogo (Souro Sanou National Hospital, Bobo Dioulasso, Burkina Faso); Pierre Becquart, Christian Carrière, Vincent Foulongne, Michel Segondy, and Philippe Van de Perre.(Centre Hospitalier Universitaire, Montpellier, France); Philippe Mayaud, Nicolas Nagot, and Helen A Weiss (London School of Hygiene and Tropical Medicine, London, UK).
